# Patient advocacy group involvement in health technology assessments: an observational study

**DOI:** 10.1186/s40900-021-00327-5

**Published:** 2021-11-25

**Authors:** Ann Single, Ariana Cabrera, Simon Fifer, Jane Tsai, Jin-Young Paik, Philip Hope

**Affiliations:** 1 Patient Voice Initiative, Brisbane, Australia; 2 Community and Patient Preference Research (CaPPRe), Sydney, Australia; 3 Formosa Cancer Foundation, Taipei, Taiwan; 4 Korea Kidney Cancer Association, Seoul, South Korea; 5 Lung Foundation New Zealand (Tuapapa Pukahukahu Aotearoa), Auckland, New Zealand

**Keywords:** Health technology assessment, Patient advocacy group, Decision-making, Funding, Reimbursement decisions, Patient and public involvement, Asia–Pacific

## Abstract

**Background:**

In some jurisdictions, patients and patient groups may be invited to provide input when Health Technology Assessment (HTA) is used to inform decisions about which medicines should be subsidised or funded. This input can help frame the evidence from a patient perspective, address uncertainties in the evidence and interpret it for the local setting. However, there is currently no evidence linking patient involvement with positive reimbursement decisions.

**Aim:**

We aimed to understand the expectations of patient involvement in the reimbursement process, especially among cancer patient advocacy groups (PAGs) in New Zealand (Aotearoa), South Korea and Taiwan.

**Methods:**

We developed an online survey to help understand the role that cancer PAGs play in reimbursement processes and identify knowledge gaps about the processes that might impact the efforts of PAGs. The survey elicited the views of staff and patients affiliated with PAGs (n = 43) on current practices and how the assessment and reimbursement of new cancer drugs might be improved.

**Results:**

There was variability in knowledge of the HTA assessment processes and in experience of being involved in them. Those with HTA experience were more likely to have confidence in the process. Those who had not been involved tended to have little awareness of, or frustration with, decision-making processes. Most identified cost, finances and economic assessments as key considerations in current processes. Some respondents had clear ideas about how their knowledge and involvement could improve processes to determine the value of new medicines. However, for many, a lack of information about the basis for decision making and opportunities to be involved was a barrier to identifying process improvement.

**Conclusions:**

HTA is implemented primarily in countries seeking to have fair and equitable processes for funding medicines. PAGs often recognise the financial challenges of funding new medicines and share the desire for procedural fairness. The connection PAGs make between patient involvement and improved access to new medicines may be based on the belief they can add information to the evidence base, help solve problems, ensure fairness through transparency and/or influence the culture towards increased access to medicines they value.

**Supplementary Information:**

The online version contains supplementary material available at 10.1186/s40900-021-00327-5.

## Plain English summary

**What are health technology assessments?:**

Health technologies are medicines, devices and services used to prevent, diagnose or treat medical conditions. A Health Technology Assessment (HTA) considers the value of a health technology to help governments make fair decisions about which treatments to fund or subsidise. Patients and Patient Advocacy Groups (PAGs) may be invited to provide input into this process.

**What did our study look at?:**

We surveyed staff and patients from cancer PAGs in New Zealand (Aotearoa), South Korea and Taiwan to understand their experiences and expectations of involving patients in HTA. A total of 43 people completed our survey.

**What were the results?:**

Overall, 20 of the 43 surveyed people (47%) were aware of the assessment process used to decide which medicines were funded in their country. (27 of these 50 people were PAG staff members.) Patients from South Korea and New Zealand were rarely included in the assessment process. Patient involvement was more commonly reported in Taiwan. The impact of input from patients on HTA was rated low by patients in both South Korea and New Zealand and average in Taiwan. A general lack of transparency, feedback on the process, and support from assessment agencies was also reported. Many of the patients surveyed wanted to see more patient involvement in the assessment processes.

**What is the main take-home message from this study?:**

With improved guidance and feedback from assessment agencies, PAGs and patients believe their involvement can improve decision making on treatment funding and increase fairness through transparency.

**Supplementary Information:**

The online version contains supplementary material available at 10.1186/s40900-021-00327-5.

## Background

Health technologies are interventions (including medicines) that are developed to prevent, diagnose or treat medical conditions, promote health, provide rehabilitation or organise healthcare delivery [1]. Health Technology Assessment (HTA) can be defined as ‘a multidisciplinary process that uses explicit methods to determine the value of a health technology at different points in its lifecycle – the purpose is to inform decision-making in order to promote an equitable, efficient and high-quality health system’ [1].

It is thought that HTA can be strengthened by including the patient perspective, with some countries having taken significant steps to involve patients in their HTA processes [2] and incorporate their perspectives into decision-making [3, 4].

The goals for patient involvement in HTA, first described by Abelson et al. [5], include: achieving more transparent and legitimate decisions; taking a more comprehensive approach to value determination that is informed by patients’ perspectives and lived experiences; making better decisions across all stages of the HTA process; and building capacity for patients to contribute [5].

Patient involvement in HTA can help highlight areas of unmet need, identify outcomes that matter to patients, and improve understanding of unintended or indirect impacts of health technologies [6]. For example, Berglas et al.’s 2016 descriptive analysis of 30 drug reviews by the Canadian Agency for Drugs and Technologies in Health (CADTH) Canadian Drug Expert Committee [7] identified that patient insights were used to frame HTAs, aid interpretation of evidence and raise additional issues such as progress of recovery and sustainability of health. Livingstone et al. [8] also found patient involvement added new information to HTAs along with valued context and reassurance to committees in their evaluation of two types of HTAs at the National Institute for Health and Care Excellence (NICE). The impact of patient involvement was particularly noted in the assessment of highly specialised technologies which are conducted for ultra-orphan treatments where costs may be higher, populations smaller and uncertainties in the evidence greater [8].

Patients’ perspectives are integrated into HTA using two distinct but complementary approaches: patient-based evidence from robust research that can be critically assessed, such as patient preference studies and qualitative evidence synthesis; and participation. Participation typically includes providing input (written or verbal) or membership of an expert committee. Participation can inform value judgements at any point in an HTA and is particularly useful for understanding the consequences of using a technology in the local setting [9, 10].

Despite patient involvement being generally considered beneficial, many HTA bodies do not involve patients, and among those that do, it may not be systematic, comprehensive or meaningful [11]. Expectations of patients to be able to contribute to HTA decision making have increased in line with expectations of access to new and expensive technologies [12] but the views of patients in the Asia Pacific are less documented in the literature. New, effective cancer treatments have become available in recent years, but there are discrepancies in access across different countries and regions [13].

Our study objectives were to understand the role that patients and PAGs currently play in reimbursement processes for cancer treatments in different jurisdictions in the Asia–Pacific region; to identify important knowledge gaps in reimbursement processes and decision-making that might negatively impact the efforts of the PAGs; and to elicit views on how the assessment and reimbursement of innovative cancer treatments might be improved.

## Methods

### Survey instrument development

We developed an online survey based on findings from literature research on current reimbursement listing processes in the jurisdictions of study. This included both evaluation and decision-making, mapped by jurisdiction, along with guidance from a steering committee facilitated by the Patient Voice Initiative. The objective of the steering committee was to clarify the literature research findings and to provide additional insights required to shape the survey – especially in terms of appropriate language, questions and recruitment for the culturally diverse jurisdictions – and inform the interpretation of results.

The steering committee was comprised of panel discussion members due to speak at the International Society for Health Economics and Outcomes Research (ISPOR) Asia Pacific 2020 conference in September 2020. The panellists for the discussion session (‘*Funding high-cost innovations in Asia – Is the current HTA system adequate*’) included Philip Hope (PH) – CEO, Lung Foundation New Zealand; Jane Tsai (JT) – Vice CEO, Formosa Cancer Foundation Taiwan; and Jin-Young Paik (JYP) – Founder, Korea Kidney Cancer Association. The session moderator was Ann Single (AS) from Patient Voice Initiative, and the session was funded by a grant from MSD. Panellists reflected on the survey’s findings, including the potential reasons and consequences for patient involvement in HTA in their jurisdiction.

The panellists were given various opportunities to review the survey and identify any additional questions or areas of concern. They ensured the survey avoided questions that may have unintentionally offended or criticised PAGs achievements thus far and was sensitive to the unique culture and positioning of PAGs in each country. The survey was translated into local languages and the translations reviewed by the panellists. MSD staff from each country provided input and/or feedback on the materials where appropriate.

### Survey methods

PAGs in South Korea, New Zealand (Aotearoa) and Taiwan that have been actively engaged with cancer patient activities were approached and recruited by phone, email and LinkedIn. Invitations to participate in the survey were sent via web link. In Taiwan, JT developed a paper version of the survey to cater for older people who were less likely to access an online survey. The survey was distributed for completion to staff and members of the PAGs.

*Participants:* Eligible participants were those aged 18 years or over, who provided electronic informed consent after reading the online participant information sheet and indicating their consent by ticking a box. Participants were able to withdraw at any time (by closing the online survey window) without penalty or prejudice. Participants were excluded if they self-reported a current or prior relationship with Community and Patient Preference Research (CaPPRe) or if they worked for a pharmaceutical company.

Ethics approval was not required because the research was non-interventional, and the participants were informed, consenting adults able to withdraw at any stage.

### Survey instrument

The survey instrument consisted of screening questions on participants’ gender, age, whether they were affiliated with a cancer-based patient advocacy group, and whether they were a patient or staff member. There were questions on the role and achievements of the PAG, awareness of and experience in HTA, and the role of patients in HTA in the future. For participants who were staff members of a PAG, there were additional questions on PAG details and experience. For patient participants, there were additional questions on their experience in being involved in the assessment process for cancer treatment reimbursement. Surveys took between 15 and 20 min to complete and were conducted in September 2020.

In addition to the insights provided on PAG and patient involvement in HTA, the development of the survey itself was a key component of our study. To our knowledge, this is the first survey to be developed on this subject matter for this audience. Developing questions with appropriate language and terminology, so that all respondents were able to understand and contribute, was essential to the process, and the PAG representatives on the steering committee played an essential role in this regard. Questions were carefully phrased with the aim of capturing insights rather than testing respondents’ knowledge or recognition of HTA terminology. A step-approach was implemented, where questions were shown depending on the gauged knowledge/experience from previous questions, and open-text answers for several questions meant that pre-filled answer options did not prompt or lead respondents to answer in a certain way.

All analyses are descriptive. Guidelines for the reporting of patient and public involvement in research [14] have been followed. (See Supplementary material for the full survey and Additional file 1: GRIPP2 Short form.)

## Results

### Survey respondents

A total of 43 people took part in the online survey. Participants were from PAGs in South Korea (n = 8 [all staff responses]), New Zealand (n = 6 [staff responses]; n = 3 [patient responses]) and Taiwan (n = 13 [staff responses]; n = 13 [patient responses]).

Only complete responses were included, although there were some optional open questions that were not completed by all participants. At the request of the steering committee, demographics of respondents have not been included in this paper, to ensure that identification of survey respondents is not possible. However, respondents were affiliated with a wide range of cancers including hematological, liver, lung, kidney, breast, and colorectal cancers.

### Aims and focus of PAGs in South Korea, New Zealand and Taiwan

Participants affiliated with cancer based PAGs (both patients and staff members) were asked to describe the aims and focus of their PAG, which varied between jurisdictions. In South Korea, the main aims and focus were to improve the system and patient rights; provide correct patient information; and improve awareness of the condition in society. In New Zealand, the main aims were care, advocacy and support for patients and caregivers; education and information; and research for cure. In Taiwan, holistic care; education and empowerment of patients; and the establishment of a supportive community were the main aims.

### Achievements of PAGs

The biggest achievements of the PAG, as described by the participants from South Korea, were obtaining and expanding insurance benefits for medical treatments (drugs); resolving discrimination against cancer patients; and raising awareness for young cancer patients. In New Zealand, the biggest achievements were funding research for development of and access to medicines; empowering patients with information and a support network; and raising awareness regarding a lack of funding for immunotherapies. In Taiwan, the main achievements were providing opportunities for patients to integrate into society; kindness, support and care; and encouragement of early detection and prevention of cancers.

### Awareness of the medicine funding/reimbursement assessment process

Overall, 20/43 (47%) of respondents were aware of the assessment process used to determine funding/reimbursement (27/43 [63%] of respondents were PAG staff members).

Study respondents from all three jurisdictions noted that the key pieces of information used in the assessment process were cost, efficacy, clinical data and health professionals’ expert opinions. Respondents reported some frustration around drug pricing, and while they appreciated there were restrictions or caps to reimbursement of new drugs, many felt there was little transparency around this process. Lack of funding was often reported as a reason for rejection of medicines. PAG staff respondents in South Korea cited budgetary constraints and existing alternative treatments as reasons why some medicines were rejected by the HTA. In New Zealand, some PAG staff felt that while a lack of evidence of efficacy was cited as the reason for rejection, cost was the over-riding factor in decision making.

In South Korea and New Zealand, respondents reported that the assessment agency rarely included patients in their assessment process. Patient involvement was more commonly reported in Taiwan, with 15/26 (58%) respondents reporting patient involvement. Despite the lack of direct patient involvement, PAGs had been involved in providing input into assessment – this was more common in New Zealand and Taiwan. The most common reasons cited in South Korea and New Zealand for not providing input was that they were ‘*unaware of opportunities*’. In Taiwan, some respondents suggested it was because they lacked medical or other professional expertise.

### The role PAGs currently play in the reimbursement process in Asia–Pacific

In terms of the role PAGs usually play in Health Technology Assessments, the most common response from PAG staff members in South Korea (6/8 respondents, 75%) was that there was no involvement, but two had submitted input or information for appeals (Table 1). In New Zealand PAGS had also been involved in consultations and helping to write or disseminate patient-friendly reports. In Taiwan, PAGs were involved in more stages of the process, including membership of the expert committee, submitting input and consultation on draft advice/recommendation report (Table 1). The most common types of input provided by PAGs in the assessment process are presented in Table 2.

**Table 1:** Stage that patient advocacy group (PAG) becomes involved

Stage that PAG becomes involved	South Korea (n = 8)	New Zealand (n = 9)	Taiwan (n = 26)
Topic proposal	0	0	1
Topic selection	0	0	0
Scoping of assessment	0	0	0
Submitting input	1	2	4
Membership of expert committee	0	0	5
Presentation to expert committee or hearing	0	0	2
Consultation on draft advice/recommendation report	0	1	4
Help write or disseminate patient-friendly report of advice/recommendation	0	2	3
Submit information for appeals	1	3	2

Responses from PAG staff to ‘At what stage(s) of the assessment process used to determine which medicines are funded or reimbursed does the PAG usually become involved?’

**Table 2:** Type of input provided

Type of input provided	South Korea (n = 8)	New Zealand (n = 9)	Taiwan (n = 26)
Prepare input to the agency	1	3	2
Present to committee meetings	0	0	2
Participate as a committee member	1	0	3
Provide a statement in public meetings	0	0	1
Take part in the agency consultation meetings	0	0	2
Provide patient experts to participate in workshops and meetings	0	0	2
Provide feedback on draft recommendations	1	4	2
Participate in hearings	0	1	2
Collect and report information about members’/patients’ needs, perspectives and experiences to inform input	2	3	4
Undertake or commission research about patients’/members’ needs, preferences and experiences	0	1	4
Provide information to patients about how patients can provide input to process	1	3	2

Responses from patient advocacy group (PAG) staff to 'In what way does the PAG become involved in the assessment process (i.e. what is the type of input they provide)?’

In terms of innovative cancer medicines, the PAG staff respondents had varying amounts of experience in providing patient input for assessment processes. In South Korea, three respondents (37.5%) had experience, including delivering opinions to government agencies and the media. In New Zealand, two respondents indicated that they had experience providing patient input, including providing written submissions of patient experiences and perspectives, media stories and petitions. In Taiwan, three respondents reported experience, including convening closed-door sessions with experts and compiling academic and physician comments into a white paper.

### Purpose of patient involvement in the assessment process

There were differences in responses from the three jurisdictions in terms of the purpose of patient involvement in the assessment process. Most responses from South Korea were that the purpose of patient involvement was ‘very clear’ (7/8), whereas most responses from New Zealand were that the purpose was ‘*not very clear’* (5/9). Responses from Taiwan were mixed, with ‘*clear*’ (7/26) and ‘*don’t know*’ (7/26) being the most common, followed by ‘*not very clear*’ (5/26), ‘*somewhat clear*’ (4/26) and ‘clear’ (3/26). Patient respondents from all countries indicated that, in general, the patient experience and quality of life were the most useful inputs from patients. When asked their opinions on how much of an impact patient input has on the assessment process, using a scale where 1 = ’No impact at all’ and 10 = ’Extremely large impact’, the average rating scores were: South Korea 1.9; New Zealand 2.3; and Taiwan 5.9.

The stage(s) of the assessment process that respondents would like to see improved patient involvement in is presented in Table 3. In terms of how they hoped to see patient involvement in the HTA process evolve in the future, most respondents indicated that they would like to see more patient involvement and more impact on assessment outcomes: South Korea 7/8 (87.5%); New Zealand 5/9 (56%); Taiwan 8/26 (31.0%). When asked for ideas on how this may be achieved, respondents from all three jurisdictions suggested improving the transparency of the process and increasing (and possibly mandating) patient involvement through PAGs.

**Table 3:** Future involvement of patients in Health Technology Assessment (HTA)

Future involvement of patients in HTA	South Korea (n = 8)	New Zealand (n = 9)	Taiwan (n = 26)
Topic proposal	4	4	3
Topic selection	2	5	7
Scoping of assessment	4	5	11
Submitting input	7	6	12
Membership of expert committee	6	5	6
Presentation to expert committee or hearing	6	6	8
Consultation on draft advice/recommendation report	3	6	12
Help write or disseminate patient-friendly report of advice/recommendations	4	5	14
Submit information for appeals	4	5	7
Other	1	1	0
I don’t know	0	2	3

Responses to 'At what stage(s) of the assessment process would you like to see more patient involvement and input?’

### Staff and patient experiences of being involved in HTA

In South Korea, PAG staff reported challenges in being involved in the HTA assessment process and felt there was a ‘*lack of formal process*’ with ‘*no system in place for participation*’. Similar frustrations were voiced in New Zealand: ‘*not consulted or invited to meetings*’, ‘*lack of transparency*’ and ‘*questions not answered*’. In Taiwan, the main challenge was to ‘*ease the financial burden on cancer patients*’. Staff respondents from PAGs in South Korea and New Zealand reported that the assessment agency rarely or never provided feedback on how the PAG information was used and incorporated into decisions, although there were few responses to this question. In Taiwan, there was a much higher rate of feedback provided, with 8 of 9 staff respondents (88.9%) reporting that feedback was provided.

When asked about the type of support and guidance provided by assessment agencies, South Korean and New Zealand PAG staff indicated that there was no support or guidance, and they were dissatisfied with the level of support; those from Taiwan were unsure if there was any support. While responses from patients were varied (and no responses from South Korea were recorded), many patients indicated that support was inadequate and could be improved with transparency.

### How can assessment and reimbursement of innovative cancer treatments be improved in Asia–Pacific?

Respondents felt that patient involvement in HTA of innovative immunotherapies should be different to the usual required involvement. PAG staff in South Korea stated that while these treatments may cost more, they are effective with fewer side effects, leading to an improved quality of life. PAGs in New Zealand called for a more patient-centric approach. Overall, the responses indicated that patients should be given the opportunity to contribute to the assessment process, so that the message about what patients value was properly understood.

When asked how decisions should be made when determining the value of medicines and which medicines are funded, PAG staff members from South Korea stated that the impact on patient rights and their quality of life should be accounted for; opinions from experts and patient organizations should be listened to; and the criteria for assessment should be established through social agreement and consensus. Those from New Zealand stated that evaluations should be transparent and consider the burden of disease and equity of patient access. PAG staff responses from Taiwan were that all factors should be considered, and decisions should be based on effectiveness.

Patient PAG members from New Zealand stated that decision-making should be without bias or self-interest and based on cost–benefit ratio and standard of care. In Taiwan, patients thought decisions should be made based on clinical trial results, cost, expert evaluation and interaction with patient groups.

PAG staff from South Korea and New Zealand indicated that assessment processes had not been set up to appropriately assess the value of innovative immunotherapies, with the main reason cited as prioritizing cost over effectiveness in both jurisdictions. Additional reasons given included different characteristics of immunotherapies leading to unfair assessments and dependence on large clinical trials, which are not always available for innovative targeted therapies. PAG staff members felt that the involvement of patients and patient groups in the process would improve the system to allow better access to immunotherapies for cancer patients.

## Discussion

### Summary of evidence

Patient involvement in HTAs can complement the clinical and economic evidence that HTA bodies evaluate by providing unique patient insights [3, 15]. Indeed, health authorities may be able to make more efficient and effective decisions when it comes to pricing and reimbursement when an understanding of the real-world benefits and harms of treatments are integrated with traditional evidence [3]. However, in our study, the impact of patient input on the assessment process was rated as low by patients in both South Korea and New Zealand and average in Taiwan. Many of the patients surveyed wanted to see more patient involvement and impact on outcomes through improved transparency and mandating of patient involvement in the assessment processes.

While studies of other HTA systems suggest patients add important context and additional information and issues, our survey revealed that some patients and PAG staff members currently had limited involvement in and understanding of their local HTA processes. The variation in experiences was further described when members of the steering committee reflected on the survey results when presenting at ISPOR. For example, in Taiwan, PAGs reported more opportunities for involvement and knowledge of processes and during the panel, JT explained that the Formosa Cancer Foundation in Taiwan is training other PAGs about their local processes and arming them with the tools to become active participants in the process. Such work combined with Taiwan’s inclusion of patient involvement in HTA since 2015 [16] may account for the increased awareness, involvement and impact reported by respondents in Taiwan.

A general lack of transparency, feedback on the process, and support from the assessment agencies was also reported in our survey. Also, the valuation process regarding innovative cancer immunotherapy treatments was seen as unfair because standard practice does not cater for the nuances of these new medications, resulting in an incomplete assessment of their benefits. At the panel, PH linked transparency to trust and capacity building, stating that patients in New Zealand wanted transparency in how the [assessment] framework is applied to individual treatments.

Improved guidance and feedback from assessment agencies to both patients and PAGs is needed for patients’ voices to make an impact on reimbursement decision making. The goals of patient involvement should be prespecified, explicit and measurable, with consensus among stakeholders on defining success [17]. In addition, feedback should be taken from all involved – patients, patient groups, HTA staff and HTA advisory groups and committees – to continuously improve patient input practices and evaluate the difference it makes [18]. Formal HTA body evaluations of patient involvement in HTA, and the implementation of the resulting recommendations, appear to positively impact patients’ experiences in participating, as well as the quality of their input into HTA [17].

Finally, our survey could be used as a template for designing a quantitative survey, larger-scale questionnaires or interviews for further studies in this area. While existing heavy workloads and demands common to PAGS and differences in language, culture, time zones and experiences of HTA created some challenges, especially for timelines, the involvement of PAGs in the design and interpretation of future work would be valuable. Their expertise and experience were essential for creating an accessible survey for diverse populations and interpreting the results appropriately. The opportunity for steering committee PAGS to present their own early reflections on the results in the ISPOR panel was an important part of gaining a deeper understanding of the context and potential implications of the results. In addition, the broad range of knowledge gauged by the open text questions supports the need of conditional logic in future surveys so that participants see appropriate questions depending on their knowledge levels.

## Limitations

This study has some limitations, notably the number of respondents to the survey, which prevented statistical analysis being performed. A wider distribution of the survey to various PAGs was limited in recruitment through general awareness and the use of targeted recruitment via the panellists.

The survey was initially developed to be a semi-structured quantitative survey with open-ended text. While there were rich responses, the limited responses meant the research became more qualitative. Quantitative research to explore the issues identified with a larger sample is recommended.

The variation in PAGs and patients involved was another limitation of this study. The PAGs differed in terms of their aims and purposes, with some focussed on patient support/providing resources, while others focussed on advocacy and improved access to medicine.

## Conclusions

HTA is often promoted as an equitable and transparent way to provide universal health coverage. However, these qualities may not be evident to PAGs and patients, who see disparities in access to new medicines across different jurisdictions and have little meaningful information about how the HTA process works.

Patients and PAGs may struggle to reconcile the differences in how they value a medicine compared with the value determined by HTA bodies. Without a clear understanding of the HTA process, or information about the evidence being considered and the questions that will be asked in the process, it can be difficult for PAGs to make sense of HTA recommendations. Dissatisfaction with the process may be viewed as the emotional response of individuals to an objective scientific finding, but this criticism may not give due consideration to the role of value judgements in HTA.

The 2020 definition of HTA recognises that within the multidisciplinary process of assigning value to health technologies, processes may be equitable, but value is not neutral. Value depends on the dimensions considered and the perspective taken, which may be influenced by who is involved [1]. In short, your perspective shapes the questions you ask, the evidence you seek and consider and, ultimately, the value you determine.

Many PAGs and patients in this observational survey identified a role they could play in contributing a valid alternative perspective to HTA. They often recognised the financial challenges of funding new medicines and shared the desire for procedural fairness. The connection PAGs make between patient involvement and improved access to new medicines may be based on the belief they can add information to the evidence base, help solve problems, ensure fairness through transparency and/or influence the culture towards increased access to medicines.

## Supplementary Information

**Additional file 1:** GRIPP2 Short Form: Patient Involvement in HTA - An observation study.

## Data Availability

The datasets generated and/or analysed during the current study are not publicly available but are available from the corresponding author on reasonable request.

## Abbreviations

CaPPRe: Community and Patient Preference Research
HTA: Health Technology Assessment
INAHTA: International Network of Agencies for Health Technology Assessment
HTAi: Health Technology Assessment International
ISPOR: International Society for Health Economics and Outcomes Research
PAG: Patient advocacy group
NZ: New Zealand
