# Supplementary material for: Patient advocacy group involvement in health technology assessments: an observational study
Source: Res Involv Engagem. 2021 Nov 25;7:83. doi: 10.1186/s40900-021-00327-5 (PMC8613914; doi:10.1186/s40900-021-00327-5)
Supplement: Supplementary file 1 — Additional file 1: GRIPP2 Short Form: Patient Involvement in HTA - An observation study. [file 40900_2021_327_MOESM1_ESM.docx]

**Additional file 1: GRIPP2 Short form**

| **Section and topic** | **Item** | **Reported on page No** |
| --- | --- | --- |
| 1: Aim | Report the aim of PPI in the study | p.8 |
| 2: Methods | Provide a clear description of the methods used for PPI in the study | p.9 |
| 3: Study results | Outcomes—Report the results of PPI in the study, including both positive and negative outcomes | p.12 |
| 4: Discussion and conclusions | Outcomes—Comment on the extent to which PPI influenced the study overall. Describe positive and negative effects | p.20 |
| 5: Reflections/critical perspective | Comment critically on the study, reflecting on the things that went well and those that did not, so others can learn from this experience | p.21-22 |

PPI=patient and public involvement
